# Mannosidase activity of EDEM1 and EDEM2 depends on an unfolded state of their glycoprotein substrates

**DOI:** 10.1038/s42003-018-0174-8

**Published:** 2018-10-18

**Authors:** Marina Shenkman, Efrat Ron, Rivka Yehuda, Ron Benyair, Isam Khalaila, Gerardo Z. Lederkremer

**Affiliations:** 1 School of Molecular Cell Biology and Biotechnology, George Wise Faculty of Life Sciences, Tel Aviv University, Tel Aviv, 69978 Israel; 2 Avram and Stella Goldstein–Goren Department of Biotechnology Engineering, Ben-Gurion University of the Negev, Beer-Sheva, 84105 Israel

## Abstract

Extensive mannose trimming of nascent glycoprotein N-glycans signals their targeting to endoplasmic reticulum-associated degradation (ERAD). ER mannosidase I (ERManI) and the EDEM protein family participate in this process. However, whether the EDEMs are truly mannosidases can be addressed only by measuring mannosidase activity in vitro. Here, we reveal EDEM1 and EDEM2 mannosidase activities in vitro. Whereas ERManI significantly trims free N-glycans, activity of the EDEMs is modest on free oligosaccharides and on glycoproteins. However, mannosidase activity of ERManI and the EDEMs is significantly higher on a denatured glycoprotein. The EDEMs associate with oxidoreductases, protein disulfide isomerase, and especially TXNDC11, enhancing mannosidase activity on glycoproteins but not on free N-glycans. The finding that substrate unfolded status increases mannosidase activity solves an important conundrum, as current models suggest general slow mannose trimming. As we show, misfolded or unfolded glycoproteins are subject to differentially faster trimming (and targeting to ERAD) than well-folded ones.

## Abstract (web summary)

Marina Shenkman et al. show that the ERAD mannosidases EDEM1 and EDEM2 have bona fide mannosidase activity in vitro. The activity of these enzymes is substantially faster when their glycoprotein substrates are in the unfolded state, suggesting a mechanism for efficient ERAD targeting of unfolded or misfolded glycoproteins.

## Introduction

It is well established that extensive trimming of α1,2 mannose residues is required for the targeting of misfolded glycoproteins to ERAD in mammalian cells^1,2^, reviewed in ref. ^3–6^. Removal of three or all four α1,2 mannose residues is necessary for two purposes. One is to preclude re-addition of a glucose residue and thus remove the glycoprotein molecule from the calnexin folding cycle. The second is to allow binding to the lectins OS-9 and XTP3-B that target the glycoproteins to ERAD^7–10^. The mannose-trimming process is crucial for glycoprotein quality control, as it determines ultimate fate between productive folding or degradation. The trimming of the mannose residues is accomplished by ERManI with the help of the EDEMs^1,11,12^ and Mannosidase IA^13^. The slow activity of the mannosidases allows sufficient time for the glycoprotein molecules to fold, before their mannose residues are trimmed, which prevents their premature targeting to ERAD. This slow mannose trimming is achieved by a process of compartmentalization, in which the mannosidases are located most of the time in specialized quality control vesicles, segregated from their glycoprotein substrates^3,13,14^.

Although it was shown that overexpression or depletion of the EDEMs affects the trimming of mannose residues (EDEM1^11,15,16^, EDEM2^12^, EDEM3^17^), mannosidase activity has not yet been established in vitro for EDEM1 and EDEM2. Mannosidase activity has already been determined in vitro for ERManI and mannosidase IA and very recently for EDEM3^18^. It has also been reported for the *Saccharomyces cerevisiae* EDEM homolog Htm1, which functions in a complex with protein disulfide isomerase (PDI)^19–22^. Proof of in vitro activity is crucial to establish that the protein is a bona fide mannosidase and does not act by modulating the activity of the other enzymes. Here, we investigate EDEM1 mannosidase activity in mammalian cells and in vitro, compared with EDEM2 and to ERManI, establishing for the first time, to our knowledge, that indeed EDEM1 and EDEM2 act independently as mannosidases, although their activity is much lower than that of ERManI. We find that the mannosidase activity of EDEM1, EDEM2, and ERManI is modulated by the folding state of the glycoprotein substrate, which has important implications for the decision-making process in glycoprotein quality control.

## Results

### EDEM1 in mannose trimming and targeting to OS-9 and XTP3-B

**Fig. 1:**
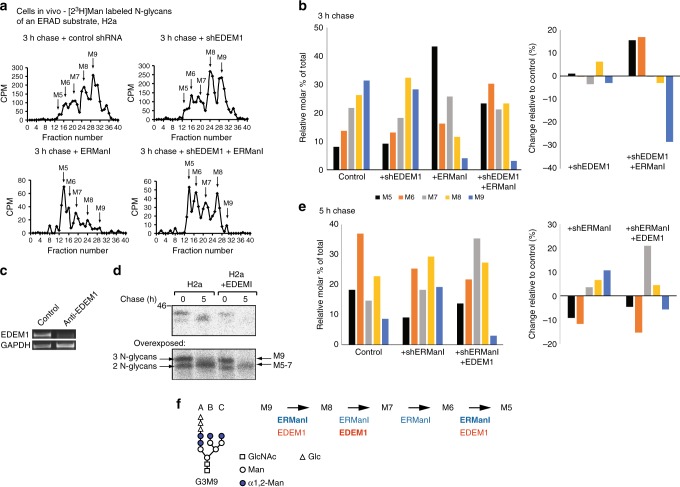
EDEM1 is required for efficient trimming to Man_5-7_GlcNAc_2_ on an ERAD substrate and can yield Man_7_GlcNAc_2_ even upon ERManI knockdown. **a** HEK 293 cells expressing uncleaved precursor of asialoglycoprotein receptor H2a together with ERManI, control anti-lacZ, or anti-EDEM1 shRNA as indicated were subjected to pulse–chase analysis using 2-[^3^H]Man followed by immunoprecipitation of H2a, release of N-glycans with endo-H and their fractionation by HPLC. M9 to M5 stand for Man_9_GlcNAc_2_ to Man_5_GlcNAc_2_. Peaks and shoulders at higher fractions than M9 are Glc_1-3_Man_9_GlcNAc_2_. **b** Relative molar amounts of each oligosaccharide species in **a** were calculated based on mannose content. The graph shows percentage of each species relative to the sum of all species present. To better visualize small changes, the right panel shows change relative to control (percent of each species in the control subtracted from the same species in each sample). **c** In parallel, RNA was extracted 48 h post transfection from HEK 293 cells expressing control anti-lacZ shRNA (lane 1) or anti-EDEM1 (lane 2) and used for RT-PCR. **d** Pulse–chase analysis with [^35^S]cys (20 min pulse) shows an increase in degradation (upper panel) and in trimming (lower panel, overexposed) of H2a upon overexpression of EDEM1-HA, compared to control GFP. After the pulse two bands can be seen, the lower one corresponding to an underglycosylated species. Mannose trimming shifts the fully glycosylated species after chase to a faster migration, whereas the underglycosylated species is quickly degraded^2^. A molecular mass marker is indicated on the left in kDa. **e** Similar to **b**, but with cells expressing H2a and control or anti-ERManI shRNA together with EDEM1 overexpression where indicated. All results shown in this figure are representative of three repeat experiments. **f** Scheme showing mannose trimming from M9 to M5. Steps where EDEM1 and ERManI appear to be involved are indicated, with their main participation in bold. The structure of the precursor G3M9 is shown, indicating branches A, B and C

To better understand the role of EDEM1 in the removal of mannose residues from misfolded N-linked glycoproteins and their targeting to ERAD, we analyzed directly the effect of EDEM1 knockdown on the N-glycans of an established model ERAD substrate, the uncleaved precursor of asialoglycoprotein receptor H2a^23,24^. This was done by pulse–chase analysis with [2-^3^H]Man labeling. The precursor of H2a is a type 2 membrane glycoprotein that is expressed endogenously only in hepatocytes. When expressed in other cell types, most H2a precursor molecules remain uncleaved, retained in the ER and are targeted to ERAD^23,25^. We had shown before the profile of N-glycans of H2a^1,2^. N-linked high-mannose glycans were separated with endo-H from immunoprecipitated H2a, and analyzed by HPLC, showing that the main effect of EDEM1 knockdown was a reduction in the mannose residue removal from Man_8_GlcNAc_2_ (M8) to Man_7_GlcNAc_2_ (M7) (Fig. 1a–c). When in combination with ERManI overexpression, EDEM1 knockdown reduced the trimming of M8 to M7-5 and of M6 to M5, suggesting that endogenous EDEM1 is required for these trimming steps, which cannot be completely compensated by ERManI. Consistent with the effect of EDEM1 knockdown, we had previously analyzed the effect of EDEM1 overexpression on the sugar chains of H2a, showing that it causes mainly trimming from M8 to M7^16^. The effect of EDEM1 overexpression can also be seen by increased degradation of the ERAD substrate and a shift in H2a migration in sodium dodecyl sulfate polyacrylamide gel electrophoresis (SDS-PAGE) (Fig. 1d). As can be seen in Fig. 1e, even upon ERManI knockdown, EDEM1 overexpression can partially compensate for trimming from the fully mannosylated M9 to M7 and from M6 to M5. Altogether, these results suggest that EDEM1 has a main role in the trimming from M8 to M7, but can also participate in the removal of the first mannose residue from M9 and of the last remaining α1,2 mannose residue to render M5. ERManI has a main role in trimming from M9 to M8 as known before, but also appears to be important for the excision of the last remaining α1,2 mannose to produce M5 (Fig. 1f).

**Fig. 2:**
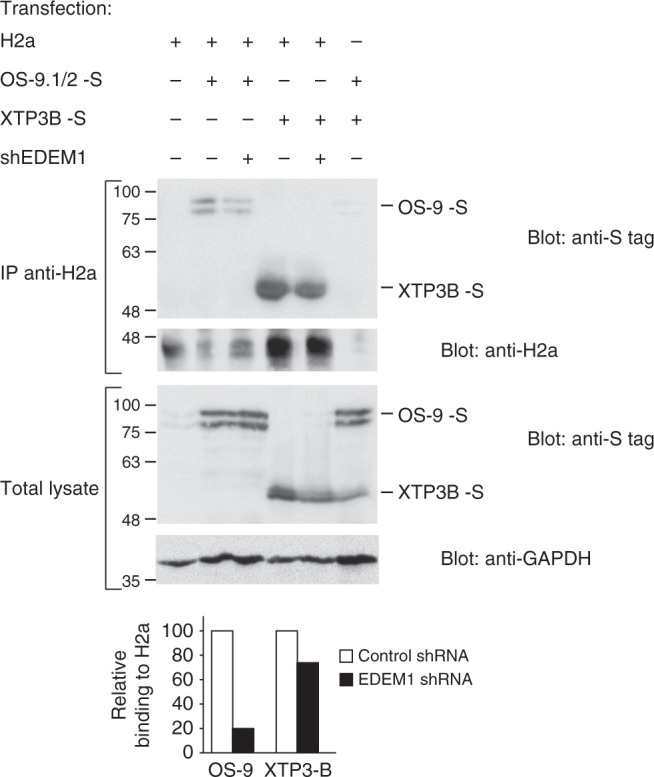
Association of the ERAD substrate glycoprotein to OS-9 requires EDEM1. HEK 293 cells expressing H2a, OS-9.1/2-S, XTP3-B-S, and anti-EDEM1 shRNA where indicated were lysed in mild conditions. Lysates (10% of total) were run on SDS-PAGE and immunoblotted with anti-S tag antibody or with anti-GAPDH (two bottom panels). The rest of the lysates were immunoprecipitated with anti-H2a, subjected to SDS-PAGE and immunoblotted with anti-S tag or anti-H2a (two upper panels). EDEM1 knockdown inhibited the association of the ERAD substrate with OS-9 by 80% and with XTP3-B by 25% (graph). The results shown are representative of three repeat experiments

We then analyzed whether the participation of EDEM1 in the mannose trimming is required for ERAD substrate association with the lectins OS-9 and XTP3-B. We had shown previously that ERManI and mannose trimming are needed for this association and for subsequent targeting to ERAD^7,16^. We determined that upon EDEM1 knockdown there was a substantial reduction in the co-immunoprecipitation (coIP) of H2a with OS-9, to 20% of the control, although we observed less effect on the association with XTP3-B (Fig. 2).

### Low in vitro mannosidase activity of EDEM1 on free N-glycans

In light of the results of the experiment in Fig. 1e, the involvement of EDEM1 in the mannose-trimming process appears to be independent of ERManI. This suggests that EDEM1 does not act as a cofactor of ERManI but probably directly as a mannosidase. A direct mannosidase activity can only be shown in vitro, and has not been reported before for EDEM1. Given the difficulties encountered by other labs in obtaining a functional recombinant EDEM1 in bacteria, we decided to immunoprecipitate EDEM1 from human embryonic kidney (HEK) 293 cells, and compare its activity with that of ERManI and with similarly processed samples but from cells expressing GFP as a negative control. Both EDEM1 and ERManI were HA-tagged so we could use the same anti-HA antibody and procedure for both proteins. As substrates we used N-linked oligosaccharides separated with PNGase F from *Macrobrachium rosenbergii* vitellogenin, because this glycoprotein displays the oligosaccharides M9 to M5, as determined previously^26^, providing alternative glycan species for the mannosidases to be tested. It is important to point out that all in vitro studies were done in blind assays, treatments being performed in one laboratory (G.L.) and samples analyzed in another (I.K.).

**Fig. 3:**
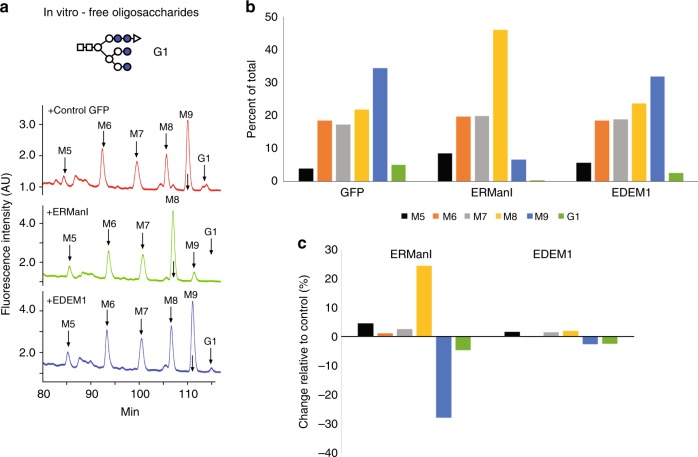
EDEM1 has a very low mannosidase activity on free oligosaccharides in vitro, compared with the activity of ERManI. **a** Scheme of a representative oligosaccharide species, G1 (Glc_1_Man_9_GlcNAc_2_) (Top). HPLC chromatograms of an oligosaccharide mix, released from *M. rosenbergii* vitellogenin with PNGase F and treated in vitro with ERManI-HA, EDEM1-HA or GFP expressed in HEK 293 cells and immunoprecipitated with anti-HA. G1 is trimmed mainly to Glc_1_Man_8_GlcNAc_2_, which here overlaps with M9, whereas Glc_1_Man_7_GlcNAc_2_, overlaps with M8. The results shown are representative of four independent experiments. **b** The chromatograms in **a** were quantified and the amount of each species was plotted as percent of total oligosaccharides. **c** The change relative to control was calculated (percent of each species with GFP **b** subtracted from the percent of the same species treated with the mannosidase)

As expected, ERManI efficiently trimmed M9 to M8. It also trimmed G1M9 to G1M8 and G1M7, which likely overlap with M9 and M8 respectively, but would not have a high contribution, given the low initial amount of G1M9. There was also a low extent of further trimming to M7–M5 (Fig. 3). Only a very low but reproducible activity was detected for EDEM1, also yielding M8–M5 from M9 and G1. (Fig. 3c).

### Low in vitro mannosidase activity of EDEM2

**Fig. 4:**
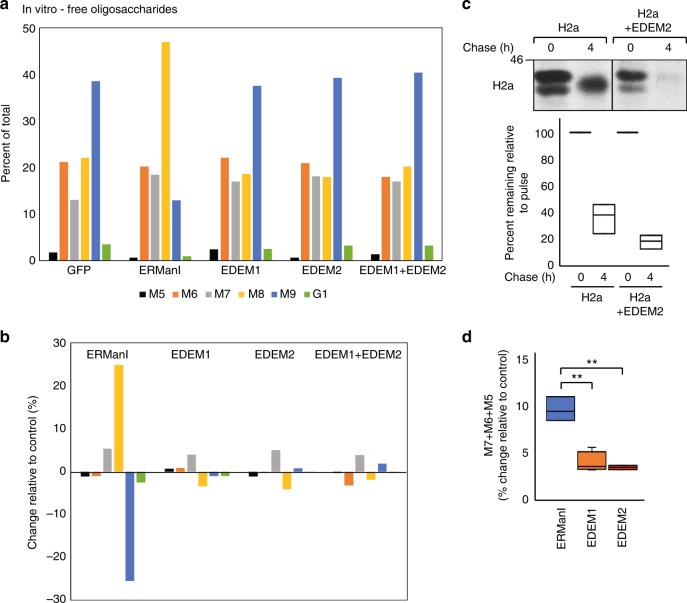
EDEM2 shows low activity in vitro on free N-glycans. **a**, **b** Similar to Fig. 3b, c but following treatment in vitro with ERManI-HA, EDEM1-HA, EDEM2-HA, EDEM1-HA together with EDEM2-HA or GFP. **c** Pulse–chase analysis in HEK 293 cells shows increased degradation, but not trimming of H2a upon overexpression of EDEM2-HA, compared with control GFP. The graph shows percentage of H2a remaining after chase relative to the pulse, from phosphorimager quantitations of three independent experiments. **d** The addition of changes relative to control for M7, M6, and M5. The graph shows data for treatment of free N-glycans with ERManI, EDEM1, or EDEM2 (three independent experiments for ERManI and EDEM1 and two for EDEM2). (*P values ERManI vs. EDEM1 0.0015, ERManI vs. EDEM2 0.008, Student’s *t* test (unpaired, two-tailed)). ERManI shows about threefold more trimming to small oligosaccharides on free N-glycans than EDEM1 or EDEM2

A similar experiment was done with HA-tagged EDEM2. A very modest activity was detected in vitro for EDEM2, trimming mainly from M8 to M7, similarly in this experiment to EDEM1 (Fig. 4a, b). The result was similar when EDEM1 and EDEM2 were combined. Despite this modest activity of EDEM2, in cells its overexpression considerably increased ERAD of H2a, as observed in pulse–chase analysis (Fig. 4c).

We compared the overall in vitro activity of extensive mannose trimming by ERManI, EDEM1, and EDEM2, measured as the percent of change relative to the control in the sum of the trimming to smaller glycans (M7-5). The trimming to M7-5 determines targeting to ERAD^1–3,5^. Although there were differences between experiments in the extent of trimming to individual oligosaccharide species, when analyzing the overall extensive trimming to M7-5 the experiments could be averaged and the changes were highly significant (Fig. 4d). Extensive trimming by ERManI compared with control was threefold higher than that by EDEM1 or EDEM2.

### In vitro mannosidase activity on glycoproteins

**Fig. 5:**
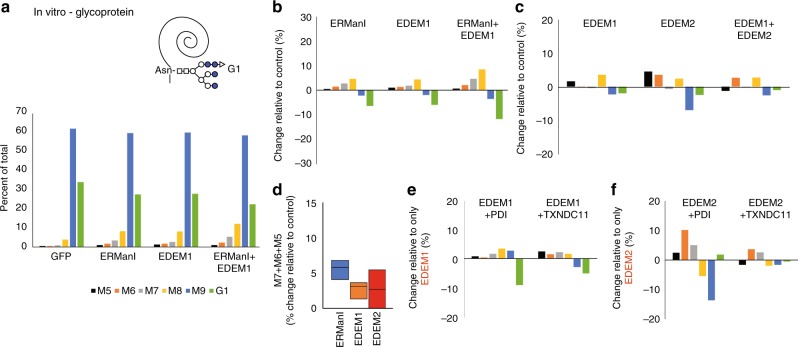
EDEM1, EDEM2, and ERManI have very low mannosidase activity on glycoproteins in vitro. EDEM1 and EDEM2 activities are enhanced by oxidoreductases. **a**–**c** Scheme of a representative N-glycan, G1, linked to native vitellogenin (Top). The experiment is similar to that in Fig. 3b, c but following treatment in vitro of vitellogenin with ERManI-HA, EDEM1-HA, EDEM2-HA, or EDEM1-HA together with EDEM2-HA. **d** The addition of changes relative to control for M7, M6, and M5. The graph shows data for treatment of vitellogenin with ERManI, EDEM1, or EDEM2 (three independent experiments for ERManI and EDEM1 and two for EDEM2). **e**, **f** Similar to **b**, but in this case EDEM1-HA or EDEM2-HA were combined with the oxidoreductases FLAG-PDI or FLAG-TXNDC11 and the change was calculated relative to the treatment with only EDEM1 or only EDEM2 instead of GFP

To test whether the protein moiety influences the mannosidase activities, we used as substrate vitellogenin. Surprisingly, ERManI showed decreased activity compared to that on the free oligosaccharides (Fig. 5a, b compared with Figs. 3, 4). EDEM1 also showed a modest activity, similar to that of ERManI, trimming from G1 and M9 mainly to M8 and to a lesser extent to the smaller N-glycans (Fig. 5a, b). ERManI showed an additive effect together with EDEM1, with increased production of M8 and M7 (Fig. 5a, b). EDEM2 also had low mannosidase activity on the glycoprotein and no additive effect when combined with EDEM1 (Fig. 5c). When comparing the overall extensive mannose trimming to M7-5, ERManI showed a higher activity than EDEM1 or EDEM2 but the difference was not significant (Fig. 5d).

As the *S. cerevisiae* EDEM homolog Htm1p was shown to associate with PDI to produce a functional mannosidase^19,21,22^, we tested whether mammalian PDI would increase the activity of EDEM1. Indeed, we could see an increase in trimming, although modest, when EDEM1 was in the presence of PDI (Fig. 5e). The protein TXNDC11 was recently reported to be a disulfide reductase with a role in ERAD^27^. Therefore, we tested whether it might also affect the activity of EDEM1. Similar to the effect of PDI, TXNDC11 modestly increased the trimming by EDEM1 of G1 and M9 to the shorter glycans (Fig. 5e). We also assessed the effect of other ER proteins implicated in ERAD, ERDJ4^28^, and ERDJ5, both J domain containing proteins, and in the case of ERDJ5 with the additional presence of thioredoxin-like domains, a reductase activity and which interacts with EDEM1^29–31^. In this case, no increase in EDEM1 trimming activity could be observed, neither on the glycoproteins nor on free oligosaccharides when in the presence of ERDJ4 or ERDJ5 (Suppl. Figure 1). When tested on free oligosaccharides, there was no stimulation of the activity of EDEM1 by PDI either (Suppl. Figure 2).

We tested in a similar manner the effect of PDI and TXNDC11 on the activity of EDEM2. In this case, TXNDC11 also had an effect, but PDI showed a much stronger effect in increasing the in vitro activity of EDEM2 (Fig. 5f).

**Fig. 6:**
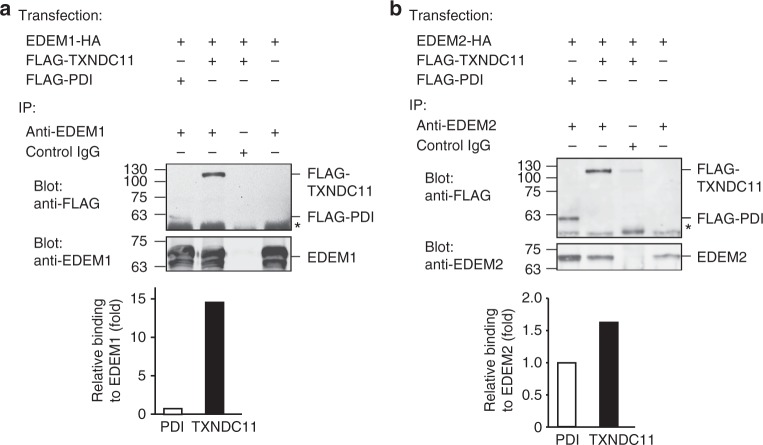
EDEM1 and EDEM2 associate with the oxidoreductases PDI and TXNDC11. **a** HEK 293 cells expressing EDEM1-HA, together with FLAG-TXNDC11 or FLAG-PDI as indicated were lysed in mild conditions. Lysates were immunoprecipitated with anti-EDEM1 or control antibody (lane 3), subjected to SDS-PAGE and immunoblotted with anti-FLAG or anti-EDEM1. The asterisk indicates a non-specific band of antibody heavy chain. Quantitation showed 15-fold stronger coIP of EDEM1 with TXNDC11. The results shown are representative of three repeat experiments. **b** Similar to **a** but with EDEM2 instead of EDEM1. Quantitation showed a moderately stronger association of EDEM2 with TXNDC11 than with PDI

We then analyzed whether EDEM1 and EDEM2 form complexes with PDI or with TXNDC11. A coIP experiment showed a robust interaction of EDEM1 with TXNDC11 and a weak but reproducible interaction with PDI, almost 15-fold weaker than with TXNDC11 (Fig. 6a and Suppl. Figure 3). A similar experiment with EDEM2 showed interaction with both PDI and TXNDC11, ~ 50% stronger with the latter (Fig. 6b and Suppl. Figure 3).

Altogether, the results suggest that EDEM1 and EDEM2 possess a very low mannosidase activity on glycoprotein substrates, which is enhanced by the action of the oxidoreductases PDI and TXNDC11. The oxidoreductases form complexes with EDEM1 and EDEM2, EDEM1 having high preference in the association with TXNDC11.

### Increased mannosidase activity on a denatured glycoprotein

**Fig. 7:**
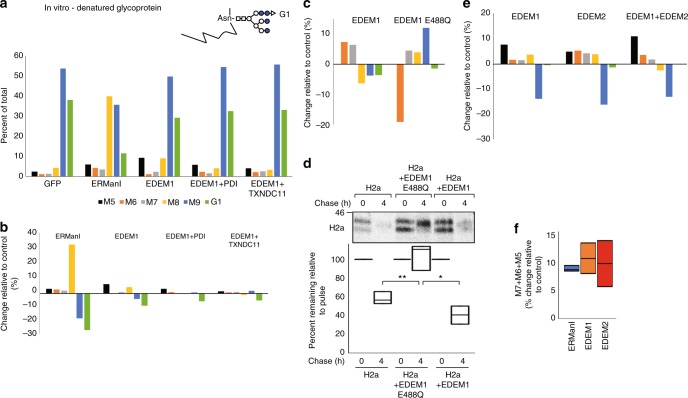
ERManI, EDEM1, and EDEM2 have increased mannosidase activity in vitro on a denatured glycoprotein. **a**, **b** Scheme of a representative N-glycan, G1, linked to denatured vitellogenin (Top). The experiment is similar to that in Fig. 3b, c but following treatment in vitro of reduced GdnHCl-denatured vitellogenin with ERManI-HA, EDEM1-HA, or EDEM1-HA together with FLAG-PDI or FLAG-TXNDC11. **c** Similar to **b**, but in this case denatured vitellogenin was treated with EDEM1-HA or with the HA-tagged EDEM1 putative binding site mutant E488Q. **d** Pulse–chase analysis of H2a in HEK 293 cells shows inhibition of degradation and of trimming of H2a upon overexpression of EDEM1 E488Q-HA. The graph shows percentage of H2a remaining after chase relative to the pulse, from phosphorimager quantitations of three independent experiments. (*P values H2a vs. H2a + EDEM1 E488Q 0.007, H2a + EDEM1 E488Q vs. H2a + EDEM1 0.015, Student’s *t* test (unpaired, two-tailed) **e** Similar to **b**, but in this case denatured vitellogenin was treated with EDEM2-HA compared with EDEM1-HA or with combined EDEM1-HA + EDEM2-HA. **f** The addition of changes relative to control for M7, M6, and M5. The graph shows data for treatment of denatured vitellogenin with similar results with ERManI, EDEM1, or EDEM2 (three independent experiments for ERManI and EDEM1 and two for EDEM2)

Given the effect of the oxidoreductases, we hypothesized that they might have influenced the conformation of the substrate. Therefore, we tested how an unfolded or denatured state of the glycoprotein substrate might affect EDEM1 activity. We and others had previously seen that EDEM1 can associate with the protein moiety of substrates^16,32–34^. Indeed, EDEM1 had an enhanced mannosidase activity on denatured vitellogenin, trimming G1 and M9 to yield mainly shorter glycans (Fig. 7a–c). With the denatured glycoprotein as a substrate, there was no additional activity when EDEM1 was in the presence of PDI or TXNDC11 (Fig. 7b and Suppl. Figure 4). This is consistent with a role of these oxidoreductases in changing the conformation of the substrate when not in a completely denatured state. Interestingly, there was also an important increase in the activity of ERManI on the denatured glycoprotein, trimming to M8 to a large extent and also to M7–M5 to some degree (Fig. 7a, b).

To assess whether a residue conserved in the catalytic pocket of GH47 family mannosidases^35^ was necessary for the mannosidase activity of EDEM1, we constructed a point mutant, EDEM1 E488Q. The mutant EDEM1 did not have a mannosidase activity when compared with wild-type EDEM1, even reducing the trimming of the denatured glycoprotein (Fig. 7c). The effect of EDEM1 E488Q was then tested by pulse–chase analysis. The mutant showed a dominant negative effect in inhibiting the degradation of H2a (Fig. 7d).

EDEM2 also showed an increased activity on the denatured glycoprotein, and there was some additive effect when EDEM1 and EDEM2 were tested in combination, yielding an increased trimming to M5 (Fig. 7e).

ERManI, EDEM1, and EDEM2 showed similar activity in the overall extensive mannose trimming to M7-5 (Fig. 7f).

**Fig. 8:**
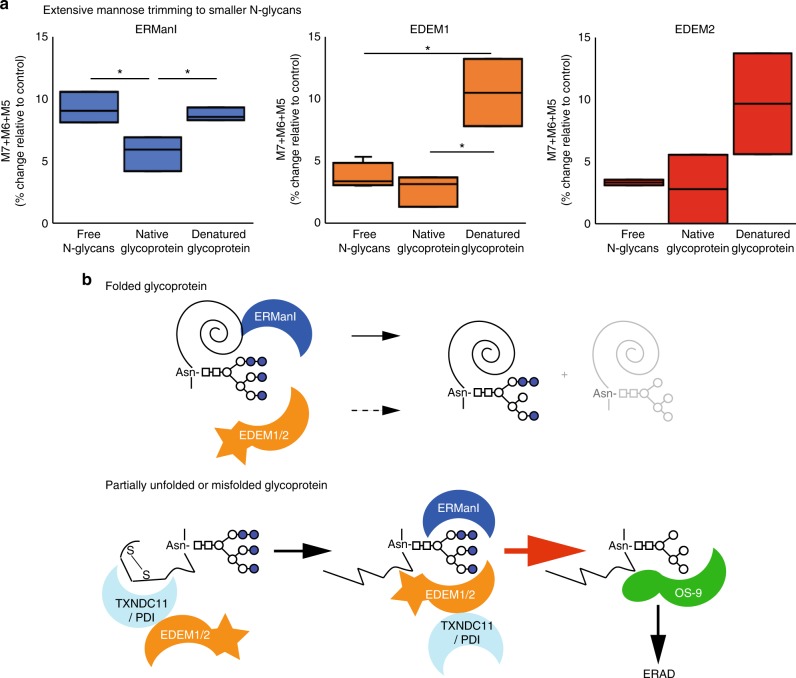
Increased mannose trimming to M7-5 by ERManI and especially by EDEM1 and EDEM2 on a denatured glycoprotein. **a** The addition of changes relative to control for M7, M6, and M5, for experiments in vitro using free N-glycans, native vitellogenin, and denatured vitellogenin. The graph shows data for ERManI, EDEM1, and EDEM2 (three independent experiments for ERManI and EDEM1 and two for EDEM2). (*P values ERManI native glycop. vs. free N-glycans 0.03, denat. vs. native glycop. 0.02, EDEM1 denat. glycop. vs. free N-glycans 0.04, denat. vs. native glycop. 0.02, Student’s *t* test (unpaired, two-tailed). **b** Working model illustrating the possible mechanism of increased mannosidase activity on partially unfolded or misfolded substrate glycoproteins. For ERManI, the folded protein moiety sterically hinders ERManI activity, leading to removal of only the terminal branch B mannose from most molecules of the native glycoprotein (upper panel), whereas the unfolded state allows engagement of the mannosidase (lower panel) and increased mannose trimming (red arrow). For EDEM1 and EDEM2 there is little or no activity on the folded molecules, whereas the unfolded state exposes determinants (possibly hydrophobic domains) on the substrate glycoprotein that allow protein–protein binding and engagement of the mannosidase with the glycans. For partially folded or misfolded glycoprotein molecules, containing disulfide bridges (lower panel), TXNDC11 and PDI, which are in complexes with EDEM1 and EDEM2, reduce the disulfides, leading to unfolding. The mannosidase activity produces M5-6, which bind to OS-9, which in turn targets the glycoprotein to ERAD. M7A (with branch A intact) could eventually bind to OS-9, but proteasomal inhibition causes accumulation of M5-6 on ERAD substrates and not M7^1,2^; M7A can still be engaged in the calnexin folding cycle

We compared the overall activity of extensive mannose trimming by ERManI, EDEM1, and EDEM2 to M7-5 on free glycans and on the native or denatured glycoprotein. In the presence of ERManI, the sum of M7, M6, and M5, representing the extensive trimming of free N-glycans was ~ 10% higher than in the control (Fig. 8a). ERManI activity was similar on the denatured glycoprotein. In contrast, ERManI activity on the native glycoprotein was significantly lower, reduced to about half. In the case of EDEM1, extensive trimming of free N-glycans was very low, ~3% change compared with control, and even lower on the native glycoprotein. In contrast, the activity of EDEM1 on the denatured glycoprotein was over threefold higher, similar in magnitude to that of ERManI (Fig. 8a). The pattern of extensive trimming with EDEM2 was similar to that of EDEM1, with low trimming activity on free glycans and on the native glycoprotein and much higher on the denatured glycoprotein, although the variability was higher and therefore the differences less significant. Altogether, these results reveal a dependence of the extensive mannose residue removal by each enzyme on the folding state of the glycoprotein substrate. This is especially evident for EDEM1 and EDEM2.

## Discussion

Previous reports, based on results of experiments performed in cells in vivo, had suggested that EDEM1 participates in the mannose-trimming process. Hosokawa et al.^11^ showed that EDEM1 overexpression increases slightly the ratio of M7A over M7C, therefore suggesting that it is involved in the trimming of the terminal mannose residue in branch C (see Fig. 1f), but trimming of mannose residues on branch A could not be excluded. In fact, an experiment of EDEM1 overexpression in mutant cells that transfer a truncated N-glycan precursor, lacking arms B and C, suggested that EDEM1 can also participate in the trimming of mannose residues from branch A^15^. The reported experiments were done upon EDEM1 overexpression and the activity of endogenous EDEM1 was not investigated. Ninagawa^12^ studied the effect of EDEM1 knockout, showing that it leads to a modest accumulation of M8. Although this was observed for total cellular glycoproteins, we see a similar effect of EDEM1 knockdown on an ERAD substrate glycoprotein (Fig. 1). Even though these results suggest that EDEM1 possesses mannosidase activity, they do not demonstrate this unambiguously. The apparent EDEM1 mannosidase activity could be dependent on endogenous ERManI or other mannosidases, whereas EDEM1 could have eventually been acting as a cofactor. A similar reasoning could be applied to results obtained with EDEM2. Knockdown and especially knockout experiments can lead to compensatory mechanisms, such as upregulation of the other mannosidases. Proof of an autonomous mannosidase activity can only be obtained in vitro, which we have tested here for the first time to our knowledge. Previous failures to obtain a functional recombinant EDEM1 might be owing to the requirement of its four N-glycans for its proper folding. We have seen that EDEM1 becomes destabilized and is quickly degraded upon cell incubation with tunicamycin (our unpublished results). Therefore, we immunoprecipitated EDEM1-HA expressed in HEK 293 cells and tested it in vitro after minimal manipulation. The isolated enzyme showed a low mannosidase activity on free oligosaccharides and on glycoproteins, but a significant increase in its activity on denatured glycoproteins. EDEM1 activity yielded trimmed N-glycans from M8 to M5 (Fig. 7). The results in cells suggest that it has some preference for trimming from M8 to M7 and from M6 to M5 and only partial redundancy with ERManI (Fig. 1). Given the preference of ERManI for the quick removal of the terminal middle branch B mannose yielding M8B, EDEM1 likely removes then an α1,2 mannose residue from branches A or C, producing M7. However, we cannot exclude some contribution of an alternative pathway where the middle branch mannose is removed last, as was revealed by differential sensitivity to mannosidase inhibitors^36^. The additive effect of combined EDEM1 and ERManI in vitro (Fig. 5), is consistent with ERManI preference for branch B mannose and EDEM1 for branches A or C. The surprising dominant negative effect of the EDEM1 mutant E488Q on ERAD suggests that, in addition to not possessing mannosidase activity, it must associate to the glycans of the substrate and inhibit activity of other mannosidases or lectins. In contrast with the results with this mutant, we had previously made an EDEM1 mutant where the complete carbohydrate recognition domain was deleted. Overexpression of this mutant did not accelerate mannose removal from an ERAD substrate. However, it still accelerated its degradation, through protein–protein interactions, bypassing the mannose-trimming requirement^16^.

The results in vitro with EDEM2 were similar to those with EDEM1, with a low mannosidase activity on free oligosaccharides and on glycoproteins and an increase on denatured glycoproteins. EDEM2 also yielded trimmed N-glycans from M8 to M5. The additive activity of EDEM1 and EDEM2 on the denatured glycoprotein, with increased trimming to M5 (Fig. 7) could suggest a complementary preference for the α1,2 mannose residues at different positions, perhaps branches A or C for EDEM1 and branch B for EDEM2, as suggested by Ninagawa et al.^12^ or alternate preferences for branches A or C by EDEM1 and EDEM2, with branch B trimmed mainly by ERManI.

We found that PDI and especially TXNDC11 associate with EDEM1 and enhance its activity on glycoprotein substrates. Conversely, especially PDI enhanced the activity of EDEM2 although it associated with both PDI and TXNDC11 (Figs. 5, 6). Because these oxidoreductases enhanced EDEM1 mannosidase activity in vitro on the native glycoprotein and not on free oligosaccharides or on the denatured glycoprotein samples, they likely act by changing the conformation of the glycoprotein. Perhaps, the substrates are not the majority of well-folded molecules but a minority of the molecules in the sample of native glycoprotein with partially folded or misfolded conformations. We could speculate that, because of conformational stress, misfolded glycoprotein molecules are more amenable to the action of the oxidoreductases, which would in turn increase the accessibility of the EDEMs to the N-glycan. This could provide a mechanism for differential enhancement of the activity of EDEM1 and EDEM2 on misfolded substrates (Fig. 8b). In contrast, mannosidase activity on completely unfolded and reduced substrate molecules (in our experiment the denatured glycoprotein sample) would not be enhanced by the oxidoreductases, because these would cause no change in accessibility of the EDEMs to the N-glycan. TXNDC11 was recently reported to be a disulfide reductase with a role in ERAD and to associate with EDEM2 and EDEM3^27^. Very recently, while this manuscript was in revision, it was reported that EDEM3 associates with another oxidoreductase, ERp46, which promotes its mannose-trimming activity^18^. In this case, the redox environment did not affect EDEM3 activity, and thus ERp46 did not affect EDEM3 per se, suggesting that the complex may reduce the misfolded substrate. There appears to be a general theme of preferential association of the mannosidases with different oxidoreductases that act on the glycoprotein substrates. It is tempting to draw a parallel to the association of calnexin and calreticulin with the oxidoreductase ERp57. Our results are consistent with those found for the *S. cerevisiae* EDEM homolog Htm1. Htm1 forms a complex with PDI, with a modest mannosidase activity and a preference for misfolded or partially unfolded glycoprotein substrates^21,22^.

We find a similar dependence for ERManI, which also shows much higher activity on a denatured glycoprotein substrate than on a native one (compare Fig. 7 with Fig. 5). This is consistent with previous findings, that suggested that the activity of ERManI is modulated by the folding status of the glycoprotein^37,38^. ERManI also acted much less efficiently on the native glycoprotein substrate than on the free oligosaccharides (compare Fig. 5 with Fig. 3). The glycoprotein used as a substrate for the in vitro studies was *M. rosenbergii* vitellogenin. A vitellogenin 3D model shows 3 exposed N-glycans^26^. ERManI can trim efficiently free oligosaccharides, so it does not appear to require hydrophobic polypeptide exposed patches. However, we can speculate that even for the exposed N-glycans the core oligosaccharide region might be more hindered from recognition than on unfolded or misfolded molecules (Fig. 8b). The results suggest low activity of ERManI, EDEM1, or EDEM2 on folded glycoproteins, even in the removal of branch B mannose, which is consistent with many glycoprotein molecules leaving the ER carrying M9. The catalytic pocket of ERManI was recently reported to interact with the complete N-glycan, including its core GlcNAc_2_^35^. In contrast to ERManI, EDEM1, and EDEM2 have very low activity on free N-glycans, hence the significant increase in their activity on a denatured glycoprotein suggests a requirement for interaction with an exposed hydrophobic domain on the glycoprotein substrate (Fig. 8).

We observed the highest in vitro activity for ERManI, much higher than that of EDEM1 and EDEM2, and the only mannosidase tested that showed considerable activity on free oligosaccharides. The group of Mori had reported a surprisingly low effect of ERManI knockout on mannose trimming^12^, which again suggests cell compensatory mechanisms for the knockout, as mentioned above. Our results suggest that ERManI, EDEM1, and EDEM2 are capable by themselves of extensive trimming to M5-6, which are the species that target a glycoprotein to OS-9, XTP3-B and to ERAD in mammalian cells in vivo^2,7,8^. However, in spite of the high mannosidase activity that we observe in vitro for ERManI, there is a relatively low amount of M5-6 produced. Trimming to M5-6 is also relatively low for EDEM1 and EDEM2. This is also true when combined, even though ERManI showed some additive activity with EDEM1 on the glycoprotein, and EDEM1 together with EDEM2 on the denatured glycoprotein. This suggests that other mannosidases may usually complete the task of trimming the N-glycan down to M5-6. EDEM1 could have an important role in this task, as its knockdown inhibited the association of the substrate with OS-9 (Fig. 2). However, there are protein–protein interactions of both EDEM1^16,32–34^ and OS-9^33^ with the substrate, which can influence this result. EDEM3 might have a role as its overexpression causes extensive trimming^17^ and its knockout was reported to lead to an accumulation of M8B^12^, but it was not determined, which species it produces in vitro. Another candidate, with much higher mannosidase activity in the production of M5-6 in vitro than ERManI, EDEM1, and EDEM2, is mannosidase IA, which we have recently shown to participate in the targeting to ERAD^13^.

Our previous findings suggested a mechanism that results in slow mannose trimming, by compartmentalization and segregation of the mannosidase from its glycoprotein substrates. Whereas the substrates are in the ER, the mannosidases are localized in quality control vesicles^13,14^. The high dependence of the mannosidase activity on the folding status of the glycoproteins, which we show here, suggests a mechanism of discrimination, by which there is faster de-mannosylation of unfolded or misfolded substrates. Upon substrate glycoprotein accumulation (ER stress), there is concentration of ERManI and EDEM1 together with the substrates at the ER quality control compartment^14,16^. Even under these conditions, ERMan1, EDEM1, and EDEM2 would have high preference for misfolded molecules.

## Methods

### Materials

Rainbow [^14^C]-labeled methylated protein standards were obtained from GE Healthcare (Little Chalfont, Buckinghamshire, UK). Promix cell-labeling mix ([^35^S]Met plus [^35^S]Cys, > 1000 Ci mmol^−1^) and Man (d-[2-^3^H(N)], 21 Ci mmol^−1^) were from Perkin Elmer-Cetus (Waltham, MA). Protein A-Sepharose was from Repligen (Needham, MA). Anti-mouse IgG agarose beads and other common reagents were from Sigma-Aldrich (St. Louis, MO).

### Plasmids and constructs

H2a subcloned in pCDNA1 was used before^25^. As described in ref. ^16^, plasmids for expression of enhanced green fluorescent protein (pEGFPN1; Clontech, Mountain View, CA) or pSUPER-retro-GFP^1^ were used as control vectors. The pSUPER vector carrying an shRNA for human ERManI, pSUPER encoding anti–human EDEM1 shRNA, pSUPER encoding anti-lacZ shRNA, and the pMH expression vector carrying HA-tagged human ERManI cDNA, EDEM1-HA in pCMVsport2, S-tagged XTP3-B and OS-9.1 and OS-9.2 were those described before^1,7,16^. EDEM1 point mutant E488Q was constructed by PCR overlap mutagenesis using the EDEM1 E488Q primers shown in Table 1. Vectors for expression of EDEM2-HA, FLAG-TXNDC11, FLAG-PDI, ERDj4-HA, and ERDJ5 were kind gifts from M. Molinari (IRB, Bellinzona), P. Lehner (University of Cambridge), E. Avezov (University of Cambridge), L. Hendershot (St. Jude Children’s Hospital, Memphis, TN), and G. Spyrou (BRFAA, Athens), respectively.

**Table 1:** Primers used in this study

Mutagenesis primers			
EDEM1 E488Q	5′-CCCTCTGAGACCACAGCTAGTGGAGTCCACATATC -3′	5′-GATATGTGGACTCCACTAGCTGTGGTCTCAGAGGG -3′	Plasmid: pCMVsport2
RT-PCR primers			
EDEM1	5′-CAATGAAGGAGAAGGAGAC-3′	5′-CAATGTGTCCCTCTGTTGTG-3′	
GAPDH	5′-CTTTTAACTCTGGTAAAGTGG-3′	5′-TTTTGGCTCCCCCCTGCAAAT-3′	
ERManI	5′-CCTTCAGTGAGTGGTTTGG-3′	5′-GTGGTCCATCTTGGCACTG-3′	

RT-PCR primers for EDEM1 and GAPDH are as described in ref. ^16^. RT-PCR primers for ERManI are as described in ref. ^1^

### Primers and reverse transcription PCR

Total cell RNA was extracted with an EZ-RNA kit (Biological Industries, Beit Haemek, Israel) and processed as in ref. ^16^, using the primers shown in Table 1 for EDEM1 and GAPDH. The primers for ERManI were those described in ref. ^1^ and are also shown in Table 1.

### Antibodies

Rabbit polyclonal anti-H2a carboxy-terminal antibodies were the ones used in earlier studies^24^ at 1:1000 for immunoblot (IB) and 1:100 for immunoprecipitation. Rabbit polyclonal anti-EDEM1 (1:1000), mouse monoclonal anti-FLAG (1:1000), mouse monoclonal anti-HA (1:1000) and mouse monoclonal anti-GAPDH (1:2000) were from Sigma, anti-S-tag (1:500) from Novagen (Gibbstown, NJ). Goat anti–rabbit, and anti-mouse IgG conjugated to horseradish peroxidase (1:10,000) were from Jackson Labs (West Grove, PA).

### Cell culture and transfections

We used the same procedure as described previously^16^. HEK 293 cells were grown in Dulbecco's Modified Eagle's Medium (DMEM) plus 10% fetal calf serum (FCS). All cells were grown at 37 °C under an atmosphere of 5% CO2. Transient transfection of HEK 293 cells was done using the calcium phosphate method. The experiments were performed 24–48 h after the transfection.

### [^35^S]Cys metabolic labeling, immunoprecipitation, SDS-PAGE

Subconfluent (90%) cell monolayers in 60-mm dishes were labeled with [^35^S]Cys, lysed, and immunoprecipitated with anti-H2 antibodies, as described previously^23,24^. In brief, cells were preincubated for 30 min with cysteine-free DMEM plus 10% dialyzed calf serum followed by labeling in the same medium containing [^35^S] cysteine (200 µCi per 0.5 ml) for 30 min at 37 °C (pulse). At the end of the pulse the cells were transferred to ice, or rinsed with the normal medium and chased for different periods of time with normal DMEM plus 10% FCS. The radioactive medium was removed and chase was commenced by adding fresh unlabeled medium supplemented with 2 mM unlabeled cysteine. At the indicated time points, the cells were cooled on ice and washed thrice with ice-cold PBS. The cells were lysed with 1% Triton X-100, 0.5% Sodium deoxycholate, lysates were incubated with anti-H2a carboxy-terminal antibody and protein A-Sepharose followed by washes of the beads with washing buffer (0.5% Triton X-100, 0.5% SDS, 0.25% Sodium deoxycholate).

SDS-PAGE was performed on 10% or 12% Laemmli gels, as described in ref. ^16^. The gels were analyzed by fluorography using 20% 2,5-diphenyloxazole and were exposed to Biomax MS film using a transcreen-LE from Kodak (Vancouver, British Columbia, Canada). Full-length gels are shown in Suppl. Figure 5. Quantitation was performed in a Fujifilm FLA 5100 phosphorimager (Tokyo, Japan).

### [2-^3^H]Man labeling and analysis of N-linked oligosaccharides

Subconfluent (90%) monolayers of cells in 100-mm tissue culture dishes were metabolically labeled for 60 min with 350 μCi ml^−1^ of [2-^3^H]Man, as described previously^1,2^. In brief, cells were rinsed and preincubated for 30 min at 37 °C with Glc-free DMEM plus 10% dialyzed FCS. They were then pulse-labeled for 60 min in the same medium containing 350 μCi ml^−1^ of [2-^3^H]Man. Cell lysis and immunoprecipitation were performed as for the [^35^S]-labeled samples. Endo-β-*N*-acetylglucosaminidase H treatment was as described before^39^. Samples were then filtered on a Centricon 30, eluted with H_2_O and the flow-through containing high-mannose N-linked oligosaccharides was separated by HPLC as described before^2,39,40^ at a flow rate of 1 ml min^−1^ in acetonitrile/1% phosphoric acid (60/40 vol/vol ratio). Fractions were monitored using a scintillation counter (Beckman Coulter, Brea, CA).

### CoIP and immunoblotting

Cell lysis was carried out in 1% NP-40, 50 mM Tris/HCl (pH 8), 150 mM NaCl, protease inhibitor cocktail (Roche) for 30 min on ice, and debris and nuclei were pelleted in a microfuge for 30 min at 4 °C, as described in^16^. The samples were immunoprecipitated with anti-H2a carboxy-terminal antibody, anti-EDEM1, or anti-EDEM2 as indicated and protein A-Sepharose or mouse anti-FLAG and anti-mouse IgG agarose beads. After overnight precipitation, the beads were washed three times with lysis buffer (diluted 1:5), which was followed by elution of the bound proteins by boiling with sample buffer containing β-mercaptoethanol at 100 °C for 5 min.

Immunoblotting and detection by ECL were done as described previously^33^ with quantitation in a Bio-Rad ChemiDocXRS Imaging System (Bio-Rad, Hercules, CA).

### In vitro mannosidase activity

Subconfluent (90%) monolayers of HEK 293 cells expressing the different mannosidases and proteins as indicated, in 2 × 150-mm dishes (~ 5 × 10^7^ cells) were lysed in 1% NP-40, 5 mM CaCl_2_, PIPES 0.1 M pH7.5, protease inhibitor cocktail (Roche) for 30 min on ice, and debris and nuclei were pelleted in a microfuge for 30 min at 4 °C. The samples were immunoprecipitated with anti-HA antibody and anti-mouse IgG agarose beads overnight at 4 °C. The immunoprecipitates were then washed twice with reaction buffer (0.2% NP-40, 5 mM CaCl_2_, PIPES 0.1 M pH7.5), which was followed by overnight incubation with *M. rosenbergii* high-density lipoprotein (HDL) containing vitellogenin or its released free N-glycans in the reaction buffer at 37 °C. To denature vitellogenin, it was treated with 6 M guanidine chloride and 20 mM dithiothreitol (DTT) for 6 h at room temperature (RT) (preceded sometimes by incubation at 95 °C), followed by addition of 200 mM iodoacetamide for 30 min at RT and overnight dialysis against reaction buffer at 4 °C.

### Purification of *M. rosenbergii* HDL containing vitellogenin

HDL purification was as described in ref. ^26^, with minor modifications. In brief, hemolymph was withdrawn from *M. rosenbergii* secondary vitellogenic females and mixed with 7% ethylenediaminetetraacetic acid in 1:1 ratio with the addition of 2 mM phenylmethylsulfonyl fluoride (Sigma). The solution was then centrifuged at 1500 g for 15 min to remove cells and debris. HDL fractions where isolated as described^41^ with slight modifications. The hemolymph solution was mixed with a saturated solution of sodium bromide to a density of 1.22 g ml^−1^. HDL containing the egg yolk protein vitellogenin was isolated by ultracentrifugation at 100,000 g for 48 h. The upper golden fraction (HDL) was collected and dialyzed against 20 mM Tris buffer pH 8.0 and stored at −70 °C until use.

### N-glycan analysis for in vitro mannosidase activity assay

After overnight treatment with the immunoprecipitated mannosidases, female *M. rosenbergii* vitellogenin was separated on SDS-PAGE. *N*-linked glycans were released from 89 kDa gel bands corresponding to the glycosylated subunit of vitellogenin according to the method described in ref. ^42^ with slight modifications as described in^26^. In brief, vitellogenin was reduced with 55 mM DTT for 10 min at 70 °C, before alkylation with 100 mM iodoacetamide at RT (protected from light). Thereafter, alkylated vitellogenin was separated on 7% SDS-PAGE and stained with colloidal Coomassie blue stain. After destaining with water, individual protein bands were excised from the gel. The gel pieces were destained with a solution of acetonitrile (ACN): 25 mM NaHCO_3_ (1:1, v:v). Subsequently, the gel pieces were dehydrated with 100% ACN and dried by vacuum centrifugation (SpeedVac). The *N*-glycans were digested in the gel with PNGase F. Glycans were released with nano-pure water followed by 100% ACN. This procedure was repeated three times. Glycans were labeled with 2-aminobenzamide by reductive amination and separated by normal-phase HPLC, with a low salt buffer system^42^. Chromatograms were quantitated using ImageJ. All chromatograms are shown in Suppl. Figure 6.

### Statistical analysis

The results of averaged experiments are presented as box plots with the box height indicating the interquartile range (IQR) in each group. The horizontal bar in the box is the median. The whiskers extend to the farthest non-outlier value smaller than 1.5 × IQR. Student’s *t* test (two-tailed) was used to compare the means of two groups. Statistical significance is indicated as *P* < 0.05 (*), *P* < 0.01 (**), *P* < 0.001 (***).

## Electronic supplementary material

Supplementary information

## Data Availability

All the data supporting the findings of this study are available from the authors upon request.
