# Supplementary material for: Mannosidase activity of EDEM1 and EDEM2 depends on an unfolded state of their glycoprotein substrates
Source: Commun Biol. 2018 Oct 18;1:172. doi: 10.1038/s42003-018-0174-8 (PMC6194124; doi:10.1038/s42003-018-0174-8)
Supplement: Supplementary file 1 — Supplementary information [file 42003_2018_174_MOESM1_ESM.pdf]

Supplementary Information

Supplementary figures

Suppl. Fig. 1. Shenkman et al

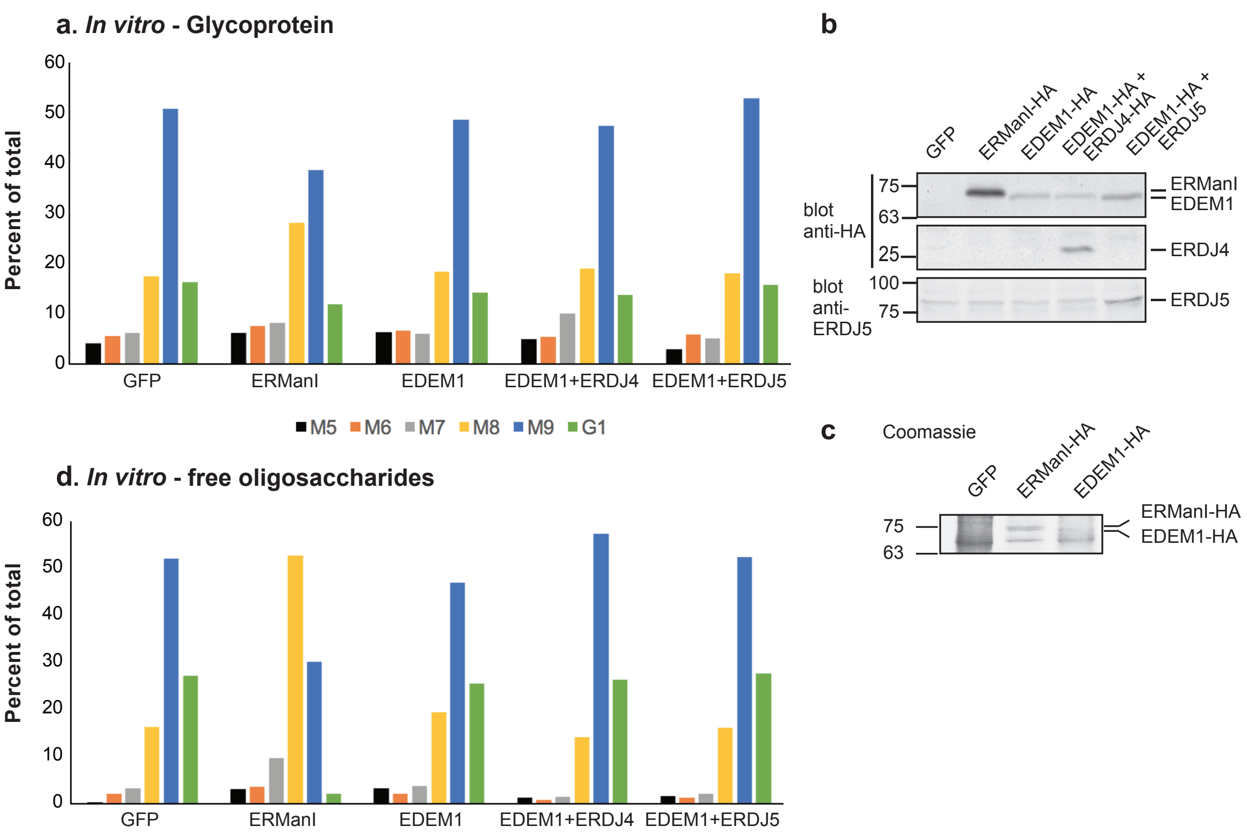

Suppl. Fig. 1. EDEM1 mannosidase activity *in vitro* is not enhanced by the presence of ERDJ4 or ERDJ5. a) Similar to Fig. 5a, vitellogenin was treated *in vitro* in this case with ERManI-HA, EDEM1-HA or EDEM1-HA together with ERDJ4-HA or ERDJ5. **b)** Immunoblots of the proteins used in the treatments in (a) with anti-HA or anti-ERDJ5 antibodies (a weak ERDJ5 signal can be observed for the endogenous protein, enhanced upon transfection with exogenous protein). **c)** SDS-PAGE and Coomassie blue staining of ERManI-HA and EDEM1-HA used in (a). **d)** Similar to (a), but using free N-glycans as substrates.

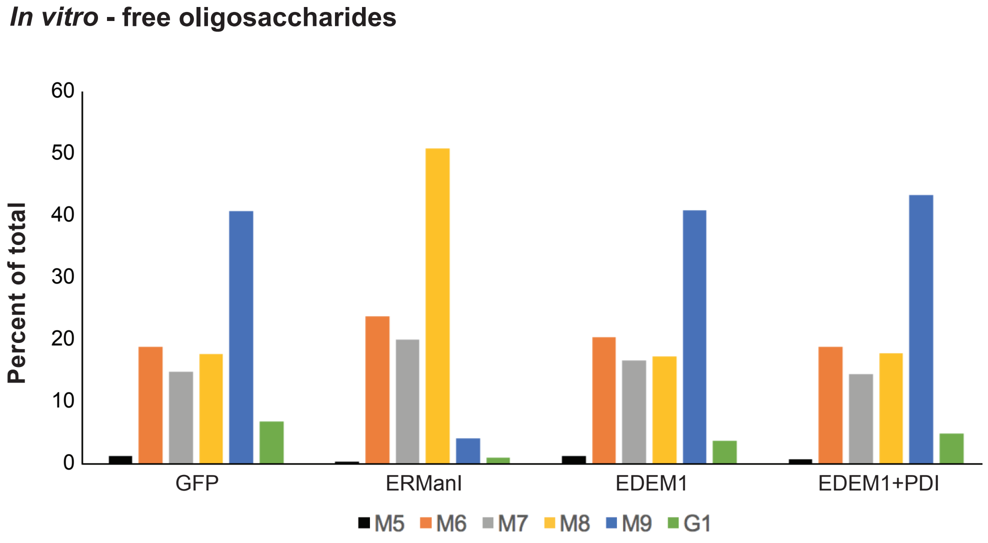

Suppl. Fig. 2. EDEM1 mannosidase activity on free oligosaccharides *in vitro* is not enhanced by the presence of PDI. Similar to Suppl. Fig. 1c but with N-glycans treated with EDEM1-HA together with FLAG-PDI.

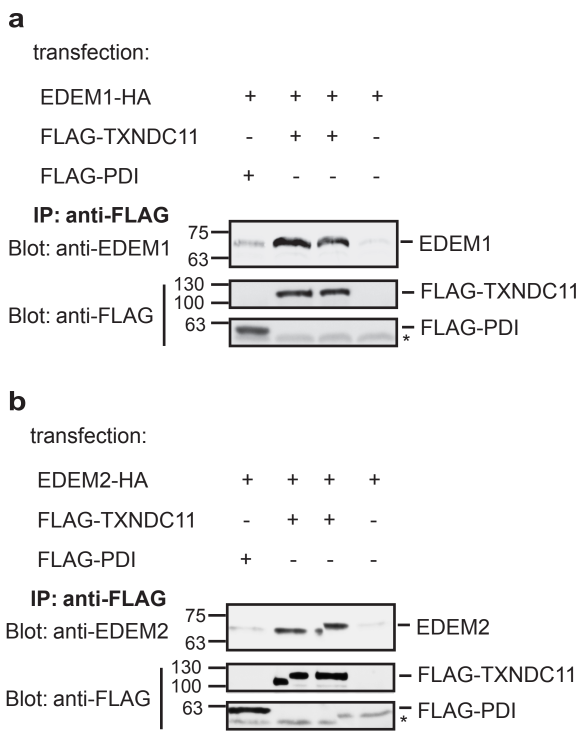

Suppl. Fig. 3. Coimmunoprecipitation of EDEM1 and EDEM2 with PDI and TXNDC11. Half of each lysate from Fig. 6a and 6b was immunoprecipitated with anti-FLAG, subjected to SDS-PAGE and immunoblotted with anti-EDEM1 (a) or anti-EDEM2 (b) or anti-FLAG (a,b). Interactions were stronger with TXNDC11 than with PDI. The results shown are representative of 3 repeat experiments.

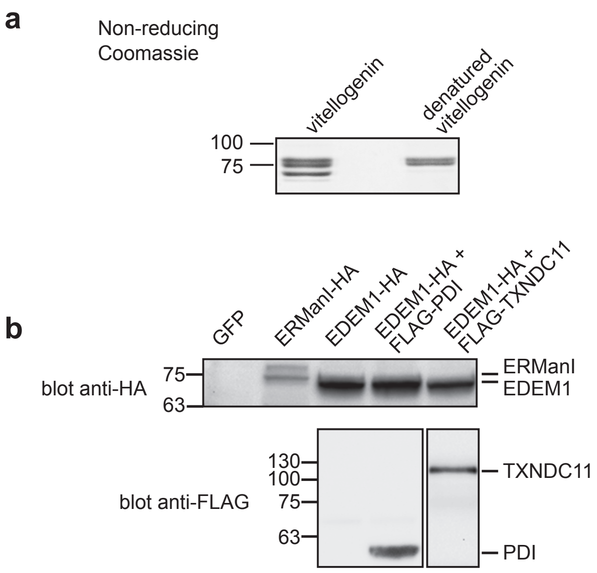

Suppl. Fig. 4. Proteins used in the treatments in Fig. 5 and 7. a) Non-reducing SDS-PAGE and Coomassie blue staining of native vitellogenin used in Fig. 5 compared to denatured vitellogenin used in Fig. 7. Faster migration of more compact disulfide-bonded molecules can be seen for the native and not for the reduced, denatured protein. **b)** Immunoblots of proteins used in Fig. 7 a, b with anti-HA or anti-FLAG antibodies. In the case of FLAG-TXNDC11 we immunoprecipitated with anti-FLAG before blotting with anti-FLAG.

Suppl. Fig. 5. Shenkman et al

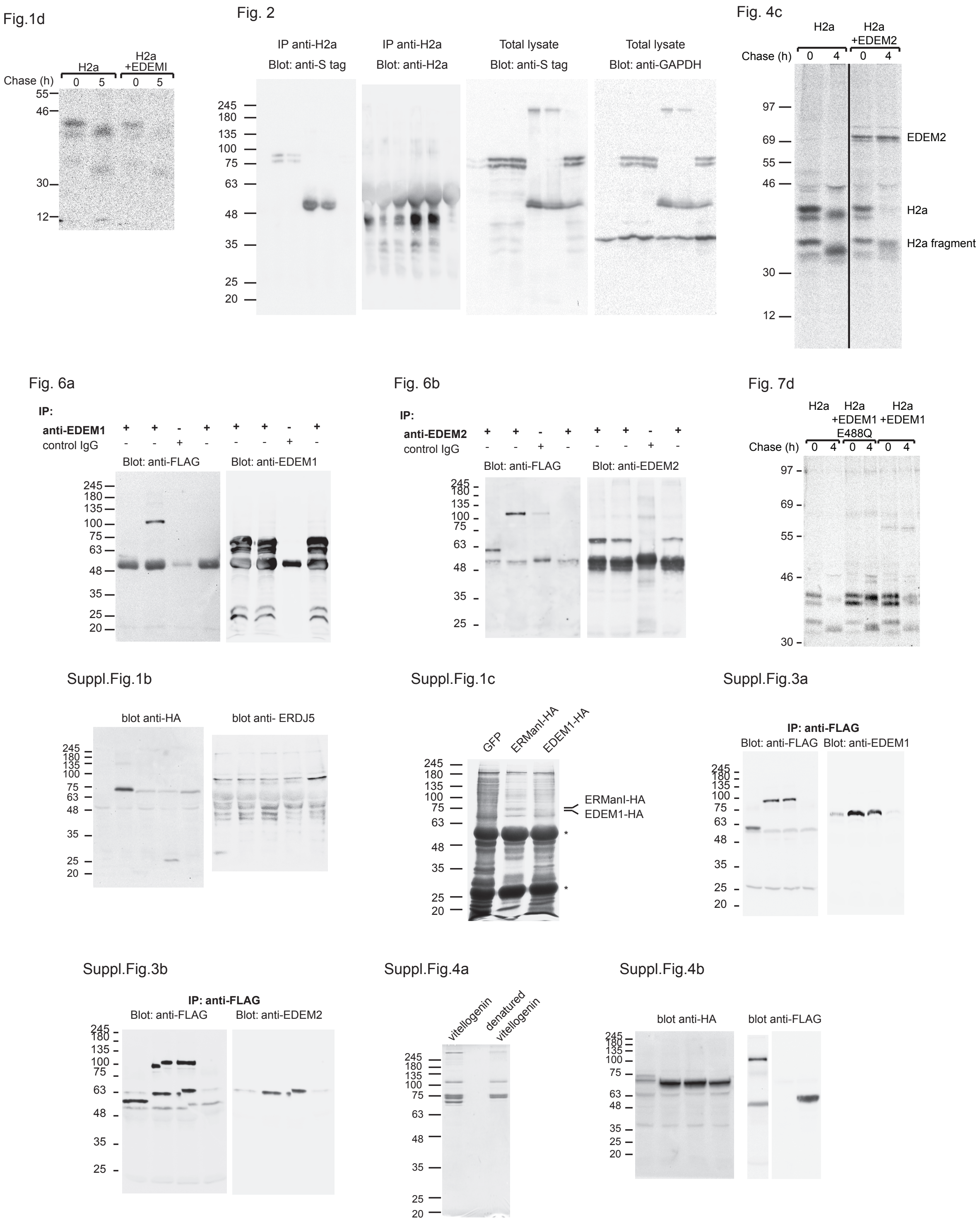

Suppl. Fig. 5. Full length gels. Full length gels and blots of all experiments in the indicated figures.

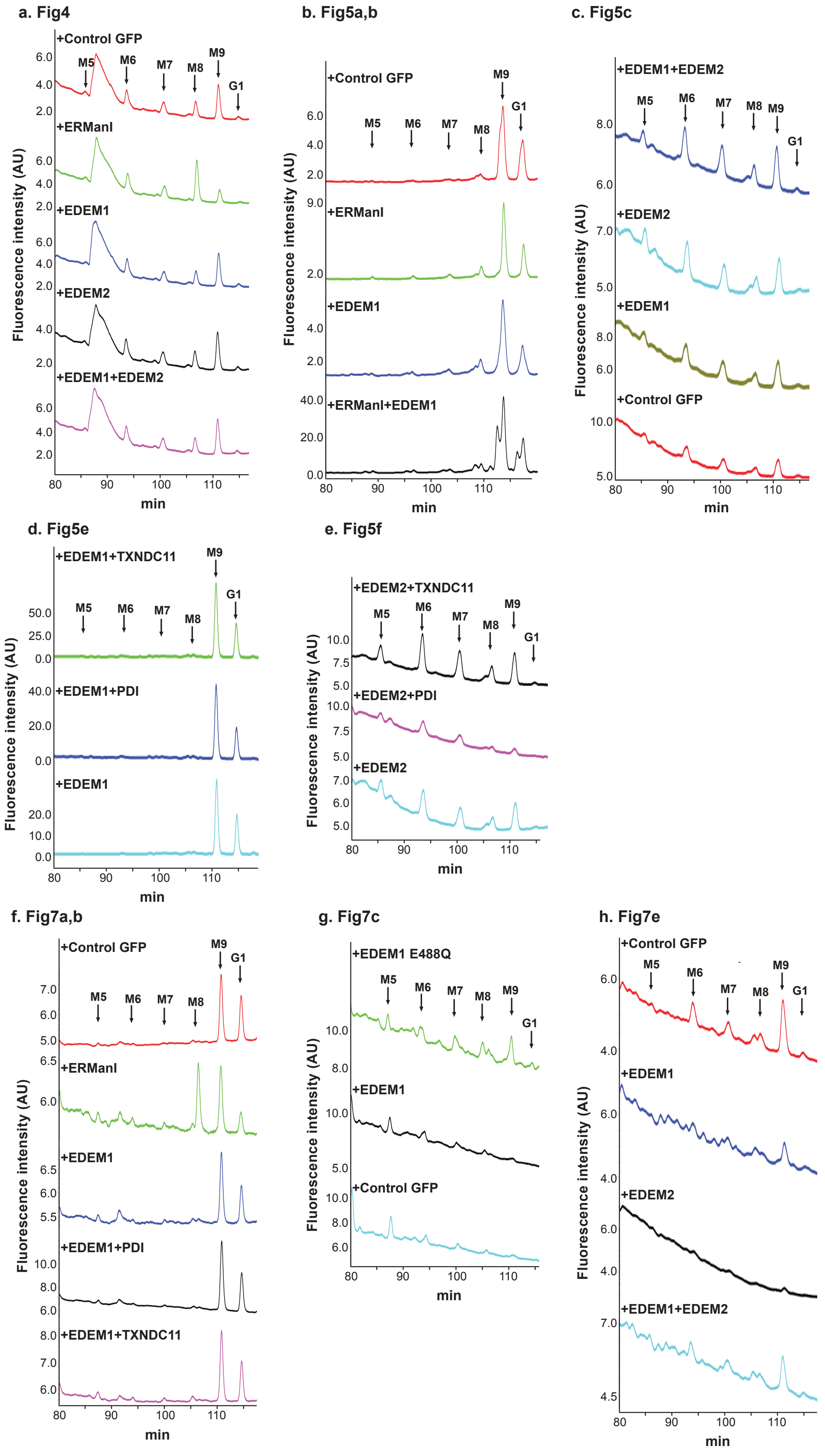

Suppl. Fig. 6. HPLC Chromatograms. All chromatograms that were quantitated and used for the graphs in the indicated figures.
